# Effect of Glycerol Pretreatment on Levoglucosan Production from Corncobs by Fast Pyrolysis

**DOI:** 10.3390/polym9110599

**Published:** 2017-11-10

**Authors:** Liqun Jiang, Nannan Wu, Anqing Zheng, Xiaobo Wang, Ming Liu, Zengli Zhao, Fang He, Haibin Li, Xinjun Feng

**Affiliations:** 1 Guangdong Provincial Key Laboratory of New and Renewable Energy Research and Development, Guangzhou Institute of Energy Conversion, Chinese Academy of Sciences, Guangzhou 510640, China; wunn@ms.giec.ac.cn (N.W.); zhengaq@ms.giec.ac.cn (A.Z.); wangxb@ms.giec.ac.cn (X.W.); liuming@ms.giec.ac.cn (M.L.); hefang@ms.giec.ac.cn (F.H.); lihb@ms.giec.ac.cn (H.L.); 2 Key Laboratory of Bio-Based Materials, Qingdao Institute of Bioenergy and Bioprocess Technology, Chinese Academy of Sciences, Qingdao 266071, China

**Keywords:** glycerol pretreatment, levoglucosan, fast pyrolysis, lignocellulose

## Abstract

In this manuscript, glycerol was used in corncobs’ pretreatment to promote levoglucosan production by fast pyrolysis first and then was further utilized as raw material for chemicals production by microbial fermentation. The effects of glycerol pretreatment temperatures (220–240 °C), time (0.5–3 h) and solid-to-liquid ratios (5–20%) were investigated. Due to the accumulation of crystalline cellulose and the removal of minerals, the levoglucosan yield was as high as 35.8% from corncobs pretreated by glycerol at 240 for 3 h with a 5% solid-to-liquid ratio, which was obviously higher than that of the control (2.2%). After glycerol pretreatment, the fermentability of the recovered glycerol remaining in the liquid stream from glycerol pretreatment was evaluated by *Klebsiella pneumoniae*. The results showed that the recovered glycerol had no inhibitory effect on the growth and metabolism of the microbe, which was a promising substrate for fermentation. The value-added applications of glycerol could reduce the cost of biomass pretreatment. Correspondingly, this manuscript offers a green, sustainable, efficient and economic strategy for an integrated biorefinery process.

## 1. Introduction

Growing global environmental concerns and the continuous depletion of fossil fuels lead to the search for sustainable alternative energy resources and technologies. Lignocellulose is the most plentiful form of biomass and the most abundant polysaccharide on Earth, which has already attracted unprecedented concern [1]. There is a widespread interest in using sugars derived from lignocellulose to produce biofuels and chemicals. Depolymerization of polysaccharides into monosaccharides is still a significant hurdle to this application [2]. Historically, research efforts on releasing sugars from lignocellulose have focused on the biochemical process and acid hydrolysis, which adopt enzymes and acids to deconstruct the lignocellulose and liberate sugars.

Fast pyrolysis is a little-explored alternative thermo-chemical depolymerization route to release sugars, which presents an excellent opportunity to establish a biorefinery [3]. Levoglucosan is the main product from cellulose fast pyrolysis, and the yield reaches as high as 59%. Levoglucosan is of great value and is mainly utilized as a chiral raw material to synthesize stereoregular polysaccharides and chiral chemicals [4,5]. Most importantly, levoglucosan can serve as feedstock for several microorganisms. Levoglucosan can be metabolized through the general glycolytic pathway after being converted to glucose 6-phosphate with the Mg-ATP-dependent levoglucosan kinase [6]. Due to the presence of levoglucosan kinase, *Aspergillus terreus*, *Aspergillus niger* CBX-209, oleaginous yeasts and engineered *Escherichia coli* KO11 can metabolize levoglucosan into itaconic acid, citric acid, lipid and ethanol with a comparable yield and rate as in the conversion from glucose [1,7,8,9,10]. As an anhydrosugar, levoglucosan can be hydrolyzed to glucose by using acid catalysts or solid acid catalysts, thereby providing a potentially rapid and efficient route to a biorefinery [11,12]. The fast pyrolysis process liberates a large amount of levoglucosan without any catalysts or enzymes in a short time, exhibiting a significant economic feasibility. It has been demonstrated that ethanol production from lignocellulose by following a combined thermochemical and fermentative approach is comparable to conventional processes [13].

The yield of levoglucosan from pure cellulose can reach as high as 59%. Nonetheless, fast pyrolysis of lignocellulose yields much lower levoglucosan than pure cellulose. Although cellulose-hemicellulose interaction is not significant, lignin inhibits the thermal polymerization of levoglucosan formed from cellulose and enhances the formation of the low molecular weight products form cellulose with reduced yield of char fraction [14]. Even small amounts of impurities can radically alter the rates of reaction and the products obtained. Alkali and alkaline earth metals (AAEMs) can shift the pyrolytic pathway of cellulose to promote the formation of ring scission products with little economic value at the expense of levoglucosan. Enhancing the selectivity of the thermochemical reactions toward depolymerization is critical to improve the conversion of lignocellulose into levoglucosan. Empirical pretreatments prior to fast pyrolysis have been performed to enhance levoglucosan yield from lignocellulose [15,16,17]. Glycerol pretreatment evidently shows an economically competitive advantage over common pretreatments, such as low boiling-point solvents, ionic liquids, hot water and dilute acid pretreatments. First, glycerol is an abundant and economic organic solvent. Furthermore, the boiling-point of glycerol is as high as 290 °C, and thus, it can be performed at atmospheric pressure. However, only a few studies have investigated glycerol pretreatment prior to fast pyrolysis. Meanwhile, a tremendous amount of glycerol presents in the liquid stream after glycerol pretreatment, resulting in a significant environmental issue and resource waste, turning into a burden for the industry. The valorization of byproduct streams of pretreatment to obtain higher value chemicals can reduce the cost of pretreatment and meet environmental requirements. Therefore, there is an urgent need to make full utilization of glycerol contained in the liquid stream from glycerol pretreatment. Till now, limited research has reported the effect of recovered glycerol from glycerol pretreatment on microbial fermentation.

**Figure 1:**
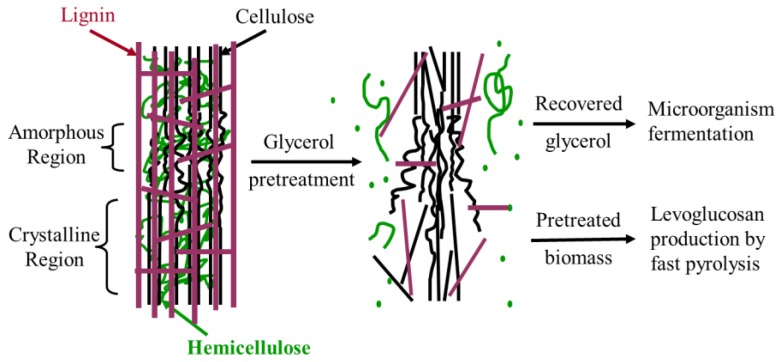
The scheme of this study.

Herein, the influences of glycerol pretreatment on subsequent glycerol fermentation and biomass fast pyrolysis were evaluated. Glycerol was comprehensively used for corncobs’ pretreatment and microorganism fermentation (Figure 1). After glycerol pretreatment, the recovered glycerol in the liquid stream was collected and further utilized for fermentation, while the pretreated corncobs were used for levoglucosan production by fast pyrolysis. A more popular configuration like the fluidized-bed reactor was not chosen because the added complexity and variability of reactor parameters were not needed in this research. The added variability would detract the focus of investigation from the effect of glycerol pretreatment on the products distribution during the fast pyrolysis process. A pyroprobe microreactor was fit-for-purpose and was therefore selected for this study.

## 2. Materials and Methods

### 2.1. Materials

Corncobs were bought from Baodi feed mill (Tianjin, China), air-dried until a constant weight and ground to pass through 80 mesh screen. Glycerol (analytical reagent) was bought from Fuyu Fine Chemical Co., Ltd. (Tianjin, China). The standard agents of xylose, glucose, mannose, arabinose, levoglucosan, 5-hydroxymethylfurfural (5-HMF) and furfural, and acetic acid were bought from Sigma-Aldrich (Shanghai, China).

### 2.2. Glycerol Pretreatment of Corncobs

For a typical pretreatment procedure, corncobs (5–20 g) were mixed with 100 g glycerol and loaded in a 250-mL round bottom three-neck flask sealed with a cork. The flask was submerged in an oil bath under vigorous stirring for 0.5–3 h with temperature control, previously heated to the desired temperature (220–240 °C). 220-0.5, 220-1, 220-2, 220-3, 240-0.5, 240-1, 240-2 and 240-3 were used to denote the pretreatment with a 5% solid-to-liquid ratio at 220 °C and 240 °C for 0.5, 1, 2 and 3 h. 240-3-5, 240-3-10, 240-3-15 and 240-3-20 were used to denote the pretreatment at 240 °C for 3 h with a 5%, 10%, 15% and 20% solid-to-liquid ratio, respectively. When the pretreatment was finished, the flask was removed from the oil bath. The glycerol pretreated corncobs were diluted with 1000 mL deionized water, then the solid and liquid fractions were separated and recovered by vacuum filtration with a 0.22-μm membrane. The solid sample was washed by deionized water to remove residual glycerol and then was dried for 24 h by a freeze dryer (Boyikang Co., Ltd., Beijing, China). The recovered glycerol contained in the liquid fraction from glycerol pretreatment was collected for fermentation.

### 2.3. Fermentation Experiments of the Recovered Glycerol

The strain utilized for fermentation was *Klebsiella pneumonia* (*K. pneumonia*). The strain was activated in Luria-Bertani (LB) at 37 °C. The LB medium was composed of NaCl (1 g/L), tryptone (1 g/L) and yeast extraction (0.5 g/L). For seed preparation, the strain was inoculated to a 250-mL flask containing 50 mL seed medium (same as the fermentation medium) at 180 rpm for 12 h. Then, the seed culture (5%, *v*/*v*) was then inoculated into the fermentation medium. Glycerol fermentation was performed in an orbital shaker at 200 rpm and 37 °C. The fermentation medium was composed of glycerol (20 g/L), citric acid (0.42 g/L), K_2_HPO_4_ (2 g/L), KH_2_PO_4_ (1.6 g/L), NH_4_Cl (5.4 g/L), MgSO_4_·7H_2_O (0.2 g/L) and trace elements solution (1 mL). The trace elements solution contained FeCl_3_·7H_2_O (0.5 g/L), ZnCl_2_ (0.684 g/L), CuCl_2_·2H_2_O (0.17 g/L), CoCl_2_·4H_2_O (0.476 g/L), Na_2_MoO_4_·2H_2_O (0.005 g/L), MnCl_2_·4H_2_O (0.2 g/L), H_3_BO_3_ (0.062 g/L) and concentrated HCl (10 mL/L). After 24 h of fermentation, the broth was centrifuged and filtered for verification. The concentrations of residual glycerol and d-lactate were analyzed by an LC-10AT High Performance Liquid Chromatography (HPLC, Shimadzu, Kyoto, Japan). A Bio-Rad column (Aminex HPX-87H, 300 × 7.8 mm, Hercules, CA, USA) was used for separation at 60 °C, and a refractive index detector (SPD-20A) was used for determination. Then, 0.005 M H_2_SO_4_ was used as the mobile phase with a flow rate of 0.6 mL/min. Dry cell weight (DCW, g/L) was tested by a UV visible spectroscopy system (Varian Cary 50 Bio, Palo Alto, CA, USA) at 650 nm. One unit of optical density was equal to 0.284 g DCW/L.

### 2.4. Elemental Analysis of Un-Treated and Glycerol Pretreated Corncobs

The organic elements were tested by a Vario EL cube analyzer (Hanau, Germany). The O content was calculated by subtracting a hundred percentage with contents of ash, C, H and N. The alkali and alkaline earth metals (AAEMs) were tested by an Optima 8000 Inductively Coupled Plasma Optical Emission Spectrometry (ICP-OES, PerkinElmer, Waltham, MA, USA). For a typical ICP-OES analysis, 0.3 g corncobs were digested by 1 mL HClO_4_ and 3 mL HNO_3_ in a test tube. The digested biomass was diluted to 10 mL using deionized water. The main analysis conditions included nebulizer flow of 1.5 L/min, flush time of 10 s, delay time of 40 s and wash time of 40 s. The normalized valencies of total AAEMs were calculated as the following equation:

(1) $$\mathrm{The}\;\mathrm{normalized}\;\mathrm{total}\;\mathrm{AAEMs}\;\mathrm{valencies}\;=\frac{(K+\mathrm{Na}+2\mathrm{Ca}+2\mathrm{Mg})\;\mathrm{in}\;\mathrm{pretreated}\;\mathrm{corncobs}}{(K+\mathrm{Na}+2\mathrm{Ca}+2\mathrm{Mg})\;\mathrm{in}\;\mathrm{untreated}\;\mathrm{corncobs}}$$

### 2.5. Compositional Analysis of Un-Pretreated and Glycerol Pretreated Corncobs

The component of corncobs was measured according to the technical report of the National Renewable Energy Laboratory [18]. After two steps of acid hydrolysis, the monosaccharides (glucose, xylose, mannose, galactose, arabinose) were analyzed by the HPLC system. An Aminex HPX-87P column (Bio-Rad, Hercules, CA, USA) was used for separation, and a refractive index detector was used for determination. Deionized water was utilized as the mobile phase, and the flow rate was set at 0.4 mL/min. The glucan content was equal to the content of cellulose, while the total content of galactan, arabinan, mannan and xylan was calculated as the content of hemicellulose. A UV spectrometer (Genesys 105, Hudson, NH, USA) was used to test acid-soluble lignin with a 320-nm wave length. Acid insoluble lignin was calculated by the difference of weight loss. The content of ash was calculated after oxidation of solid residue in a muffle furnace at 575 °C.

### 2.6. Thermogravimetric Analysis

The thermal analysis of un-pretreated and glycerol pretreated corncobs was conducted in a TGAQ50 thermogravimetric analyzer (TA, New Castle, DE, USA). Samples (6–8 mg) placed in alumina crucibles were heated from 50–750 °C at a rate of 20 °C/min. The analysis was conducted under nitrogen atmosphere with a flow rate of 20 mL/min.

### 2.7. Structural Characterization of Un-Pretreated and Glycerol Pretreated Corncobs

Fourier transform infrared spectroscopy (FTIR, Bruker TENSOR27, Optik Instruments, Brno, Czech Republic) was used to examine the main functional groups of samples. X-ray diffraction (XRD) patterns were scanned from 5°–40° with a step of 0.02° by an X’Pert PRO MPD X-ray diffractometer (PANalytical B.V., GH Eindhoven, The Netherlands). An empirical equation was used for the calculation of the crystallinity index (CrI):

(2) $$\mathrm{CrI}\;(\%)=\frac{\mathrm{ICr}-\mathrm{IAm}}{\mathrm{ICr}}\times 100\%$$

where I_Am_ was the region of amorphous cellulose and I_Cr_ represented both the region of crystalline and amorphous cellulose [19].

### 2.8. Fast Pyrolysis of Un-Pretreated and Glycerol Pretreated Corncobs

The un-treated and glycerol pretreated corncobs were fast pyrolyzed in a semi-batch CDS reactor (Pyroprobe 5200, Oxford, PA, USA). The compounds of biomass fast pyrolysis were determined by 7890A gas chromatography (GC) and 5975C mass spectrometry (Agilent Technologies, Santa Clara, CA, USA). Cellulose samples were loaded into the center of quartz tubes. The temperature of the pyro-probe was set at 500 °C. The residence time and heating rate were fixed at 20 s and 10 K ms^−1^. The temperature of the interface line was 300 °C. The split ratio was set at 50:1. Helium was used as the carrier gas. The flow rate between the pyrolyzer and GC was 20 mL/min, while the flow rate in GC was maintained at 1 mL/min. The GC column was an Agilent HP-INNO (length 30 m, internal diameter 0.25 mm, film thickness 0.25 μm). The mass spectrometer was operated in the electron impact mode (electron energy 70 eV). The oven program was 2 min at 50 °C, then 10 °C/min to 90 °C, 4 °C/min to 129 °C, 8 °C/min to 230 °C and finally held at 230 °C for 29 min. The mass was scanned from *m*/*z* 12–500 under the total ion current mode (TIC). The NIST Mass Spectral data library was used to identify compounds. Quantitative analysis of the main pyrolysis products was made by the external standard method. Five solutions of each standard compound were prepared by dissolving them in acetone. All experiments were tested in triplicate. The compound yield was calculated as:

(3) $$\mathrm{Yield}\;\mathrm{of}\;\mathrm{compound}\;(\mathrm{wt}\%)=\frac{\mathrm{mass}\;\mathrm{of}\;\mathrm{compound}\;(g)}{\mathrm{mass}\;\mathrm{of}\;\mathrm{corncobs}\;(g)}\times 100\%$$

## 3. Results

### 3.1. The Fermentability of the Recovered Glycerol

Various temperatures, ranging from 220–240 °C, and pretreatment times, varying from 0–3 h with 5–20% solid-to-liquid ratios, were tested for glycerol pretreatment. After glycerol pretreatment, the pretreated corncobs were fast pyrolyzed for levoglucosan production, while the liquid fraction from glycerol pretreatment was collected to evaluate its fermentability. A high concentration of glycerol remained in the liquid stream from glycerol pretreatment. The common microbial inhibitors, such as furfural, 5-HMF and organic acid, were undetectable in the recovered glycerol. The glycerol was recovered and utilized as the feedstock with the initial concentration of 20 g/L. The *K. pneumoniae* could metabolize glycerol to d-lactate. As shown in Table 1, 3.4–3.7 g/L DCW and 5.2–6.1 g/L d-lactate were obtained after 24 h of fermentation, with a productivity of 1.4–1.7 g/L h. Compared to the fermentation of pure glycerol, the results indicated that the cell growth and metabolism were not inhibited in the recovered glycerol. The development of synthetic biology, metabolic engineering and systems biologic tools needs to be integrated to enhance the efficiency, including product titer, productivity, yield, consumption rate and cell growth [20,21]. Considerable additional investigation is required to boost the extensive application of the recovered glycerol.

### 3.2. Main Composition and Elemental Analysis of Glycerol Pretreated Corncobs

The un-treated corncobs had a cellulose content of 32.3%; meanwhile, the hemicellulose and lignin accounted for 28.0% and 24.2%, respectively (Table 2). Glycerol pretreatment dissolved part of the lignin and hemicellulose and kept the cellulose fraction intact, leaving the solid enriched in cellulose. The glycerol showed an excellent nature and outstanding selectivity. For the pretreated corncobs (240-3), the cellulose fraction increased to 69.1%; meanwhile, the hemicellulose and lignin remained less than 10.3% and 9.2%, respectively. Further accumulation of cellulose and removal of hemicellulose and lignin occurred when longer residence time, higher temperature and a lower solid-to-liquid ratio of pretreatment were used.

The results of organic elements and AAEMs analysis of un-pretreated and glycerol pretreated corncobs are presented in Table 3 and Table 4. The C content of corncobs was affected by glycerol pretreatment. The rank order of C content of biomass constituents is lignin > cellulose > hemicellulose. Glycerol pretreatment was able to remove part of the lignin and hemicellulose. Therefore, the contents of C in pretreated corncobs were lower than that of raw material. Similarly, the contents of H and O in pretreated corncobs were higher than those of raw material. However, the glycerol pretreatment could decrease the content of N. K^+^, Na^+^, Ca^2+^ and Mg^2+^ are generally the major constituents of metal ions. The total AAEMs content of raw material reached as high as 6643.5 mg/kg. After glycerol pretreatment at 220 °C for 0.5 h, the total AAEMs content was reduced to 1518.7 mg/kg; especially, the K content declined from 4809.9 down to 190.1 mg/kg. A higher pretreatment temperature, longer residence time and lower solid-to-liquid ratio of glycerol pretreatment resulted in further removal of AAEMs. After glycerol pretreatment at 240 °C for 3 h with a 5% solid-to-liquid ratio, the lowest normalized total AAEMs valence (0.1) was achieved. For the sample of 240-3, 63.7% Mg, 66.6% Ca, 77.3% Na and 98.6% K were removed from the corncobs.

### 3.3. Mapping the Structural Changes of Corncobs after Glycerol Pretreatment

From the XRD analysis, both the raw and pretreated samples had the typical cellulose I diffraction angles. The CrI of the raw corncobs was 40.7%. After glycerol pretreatment, the CrI increased gradually, and the highest CrI value (72.9%) was achieved from the sample of 240-3 (Table 5). The dissolution/removal of amorphous hemicellulose and lignin should be responsible for the accumulation of crystalline cellulose and the increase of CrI.

**Figure 2:**
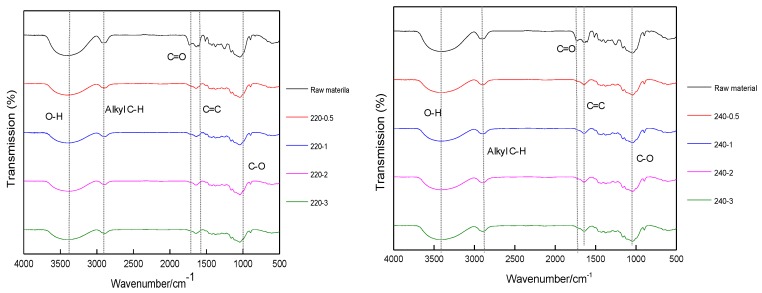
FTIR spectra of un-treated and glycerol pretreated corncobs.

Structural changes of raw and pretreated corncobs were determined by FTIR spectroscopy (Figure 2). The results illustrated that the samples before and after pretreatment had absorption at 3396–3420 and 2899–2908 cm^−1^; corresponding to the stretching of the –OH group and C–H bonds of the alkyl group, respectively. The band around 1730 cm^−1^ corresponded to carbonyl and carboxyl stretching. The bands at 1605 and 1512 cm^−1^ were from the skeletal and stretching vibration of benzene rings. Absorptions at 1370–1205 cm^−1^ were mainly from the bending of C–H or O–H bonding in polysaccharides, including cellulose and hemicellulose. The region of 1160–1030 cm^−1^ represented the bending of C–O or C–O–C stretch and deformation bands in polysaccharides and lignin. The intensified signal in this area could be attributed to the increase of the cellulose fraction. It could be indicated that after glycerol pretreatment, the carbonyl and carboxyl stretching at 1715 cm^−1^ became weak, suggesting the removal of hemicellulose. Simultaneously, absorptions at 1370–1205 cm^−1^ and 1160–1030 cm^−1^ became strong, proving the substantial increase of polysaccharides in the pretreated biomass. With the increasing glycerol pretreatment temperature and residence time, the signals related to the functional groups in lignin and hemicellulose decreased, and the signals related to the cellulose increased. These results anticipated that glycerol pretreatment could selectively breakdown chemical bonds and functional groups, thereby modifying the microstructure of biomass, resulting in the reservation of cellulose and the removal of lignin and hemicellulose.

### 3.4. Thermogravimetric Analysis of Corncobs after Glycerol Pretreatment

**Figure 3:**
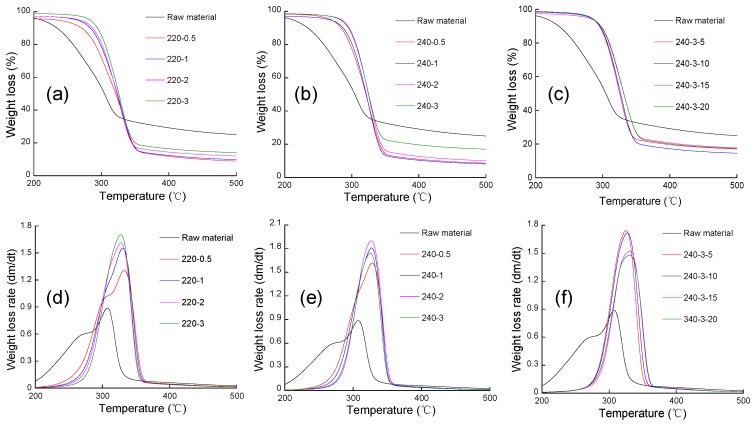
TG (thermogravimetry) (**a**–**c**) and DTG (differential TG) (**d**–**f**) profiles of raw material and glycerol pretreated corncobs.

The thermogravimetry (TG) and differential TG (DTG) curves of samples are presented in Figure 3. The DTG curve of un-pretreated corncobs showed three peaks, which was attributed to the decomposition of hemicelluloses, cellulose and lignin. In glycerol pretreated corncobs, with the removal of partial hemicellulose, the first shoulder at lower temperature gradually disappeared. According to the TGA results, the raw corncobs began to degrade at 211.2 °C (Table 5). In the case of glycerol pretreated samples, the onsets of degradation were initiated at higher temperatures, approximately 246.6–284.4 °C. The maximum degradation temperature and rate of raw material were 307.6 °C and 0.9%/min, which were obviously lower than those of glycerol pretreated, approximately 330 °C and 1.6%/min. The residuals of raw material and pretreated corncobs (240-3) were different, accounting for 19.8% and 10.3%, respectively. Those phenomena could be ascribed to the partial removal of hemicellulose and lignin and effective demineralization by glycerol pretreatment, leading to a relatively low residue and a high thermal stability. Take K^+^ as an example. The catalytic role of K^+^ has been investigated inferring that K^+^ can lower the initial decomposition temperature, decrease the maximum degradation temperature and reduce the maximum degradation rate [22]. Furthermore, biomass with higher crystallinity begins to decompose at higher temperatures, presenting sharper DTG curves and higher thermal decomposition activation energies [23,24].

### 3.5. Levoglucosan Production from Glycerol Pretreated Corncobs

Cellulose pyrolysis involves primary and secondary reactions. The initial breakage of glucosidic bonds and the formation of active cellulose occur in the primary reaction. In the secondary reaction, the active cellulose is further degraded to levoglucosan by dehydration of side functional groups [25]. The further decomposition of levoglucosan results in the formation of furfural, 5-HMF, hydroxyacetone, hydroxyacetaldehyde and some C_1_-C_2_ molecules. The yields of the main products from un-treated and glycerol pretreated corncobs’ fast pyrolysis are presented in Table 6. Compared with raw material, the glycerol pretreated samples yielded more 5-HMF and simultaneously less acetic acid and furfural. Without the pretreatment step, the un-treated corncobs released only 2.2% levoglucosan during the fast pyrolysis. Levoglucosan yields from glycerol pretreated corncobs were obviously higher than that of un-treated material. Glycerol pretreatment improved the downstream levoglucosan yield in fast pyrolysis. The further increase in pretreatment temperature and residence time had a positive influence on levoglucosan formation. The maximum levoglucosan yield (35.8%) was achieved from the 240-3 sample, which was obviously higher than that from the un-treated sample.

It was apparent that the glycerol pretreatment could enhance levoglucosan production from corncobs. The improvement mainly contributed to the enrichment of crystalline cellulose and the removal of AAEMs. The allomorph and relative crystallinity of cellulose can alter the slate and yield of compounds produced from biomass fast pyrolysis. The effect of biomass crystallinity structure on pyrolysis reactions has been investigated. It has been postulated that crystalline cellulose is favorable for levoglucosan production, while amorphous cellulose contributes more to gas and char production [26]. During levoglucosan formation, the crystalline structure is maintained, and the highly crystalline cellulose produced vapors dominated by levoglucosan [27]. Cellulose with a higher crystallinity tends to produce levoglucosan in a higher yield [24]. Previous researches have clearly demonstrated that the ash content significantly influences the yield of bio-oil and the product distribution of biomass fast pyrolysis. Inorganic compounds can also enhance the formation of char and gaseous species at the expense of anhydrosugars. In the presence of minerals, cellulose is mainly decomposed via depolymerization leading to low molecular weight products (especially glycolaldehyde) as the major products, whereas in the absence of minerals, levoglucosan is the main compound [28]. K^+^ has a catalytic effect, promoting depolymerization/fragmentation reactions for the formation of lower molecular weight oxygenates [29]. Mg^2+^ and Ca^2+^ promote the primary production of char from cellulose and the transformation of levoglucosan into furans and light oxygenates. In terms of levoglucosan yield, various cations have been examined, and the following trends have been found in the order of strongest to mildest effect: K^+^ > Na^+^ > Ca^2+^ > Mg^2+^ [30]. The result has demonstrated that even a trace level of AAEMs is sufficient to significantly alter the thermal degradation rate, chemical pathways and pyrolysis products’ distribution. Previous research showed that a very small amount of KCl (0.004 mmol/g cellulose) resulted in steep decline from 59% down to 29% of levoglucosan yield, and the presence of as little as 0.5% switchgrass ash nearly tripled the formic acid yield and quadrupled the glycolaldehyde formation, while the yield of levoglucosan reduced to less than half of that from pure cellulose [31]. It has been postulated that the activation energy for alternative pathways is diminished as a result of inorganic addition, which can promote the formation of acetol, formic acid and glycolaldehyde directly from cellulose, thereby reducing levoglucosan yield. The use of levoglucosan in bio-oil as a fermentation feedstock depends on more than just the production of bio-oil with high sugar content, but the utilization is hindered by the presence of inhibitors for the biocatalyst [32]. Considerable efforts in decreasing toxicity and enhancing biocatalyst tolerance are vital steps for biochemical utilization of lignocellulose-derived sugars.

## 4. Conclusions

Herein, glycerol was utilized in corncobs’ pretreatment prior to fast pyrolysis for levoglucosan production, and then, the recovered glycerol was further utilized for microbial fermentation to evaluate its fermentability. The results indicated that glycerol pretreatment could accumulate crystalline cellulose and remove most of the AAEMs. The levoglucosan yield from glycerol pretreated corncobs was obviously improved to 35.8% from 2.2%. Meanwhile, the recovered glycerol from glycerol pretreatment enriched in glycerol was an attractive and economical substrate for microorganism fermentation. The glycerol value was maximized, and the cost of biomass pretreatment was diminished to some extent. Consequently, this strategy also exhibited excellent potential to improve economic viability for the industrial production of levoglucosan and D-lactate and could be considered as a sustainable and promising route for a biorefinery.

## Figures and Tables

**Table 1:** The fermentation of the recovered glycerol. DCW: dry cell weight.

Substrates	Cell mass (g/L)	Glycerol consumption (g/L)	d-Lactate (g/L)	Productivity (g/L DCW)
Pure glycerol	3.7	19.6	6.1	1.7
220-0.5	3.5	18.9	6.0	1.7
220-1	3.5	18.9	6.0	1.7
220-2	3.6	18.7	6.0	1.7
220-3	3.7	19.2	5.6	1.5
240-0.5	3.5	19.1	6.1	1.7
240-1	3.4	19.6	5.8	1.7
240-2	3.7	19.3	5.3	1.4
240-3	3.5	19.2	5.7	1.6
240-3-10	3.6	18.6	6.0	1.7
240-3-15	3.6	19.1	5.8	1.6
240-3-20	3.6	19.1	5.2	1.4

**Table 2:** Recovery rate and chemical composition of glycerol pretreated corncobs.

Samples	Recovery rate (%)	Cellulose (wt %)	Hemicellulose (wt %)	Lignin (wt %)
Un-treated	—	32.3	28.0	24.2
220-0.5	78.9	38.2	26.8	20.6
220-1	64.7	50.8	20.8	16.3
220-2	53.4	60.4	15.6	12.5
220-3	49.6	64.4	13.1	10.9
240-0.5	70.5	45.1	23.4	18.4
240-1	57.2	56.3	16.9	14.7
240-2	50.6	63.8	13.4	11.5
240-3	47.5	69.1	10.3	9.2
240-3-10	49.1	65.6	11.7	10.6
240-3-15	52.3	60.5	13.4	12.5
240-3-20	54.0	56.9	16.1	14.1

**Table 3:** Organic elemental analysis of un-treated and glycerol pretreated corncobs.

Samples	C (wt %)	H (wt %)	N (wt %)	O (wt %)
Un-treated	44.6	5.9	0.3	38.7
220-0.5	44.2	6.3	0.1	41.3
220-1	43.7	6.3	0.1	42.5
220-2	41.8	6.0	0.1	44.9
220-3	43.1	6.2	0.1	44.6
240-0.5	42.8	6.3	0.1	43.8
240-1	42.1	6.1	0.1	45.2
240-2	42.7	6.0	0.1	44.9
240-3	43.1	6.0	0.1	45.0
240-3-10	43.3	6.1	0.1	44.0
240-3-15	43.6	6.0	0.1	42.5
240-3-20	43.8	6.1	0.1	42.1

**Table 4:** Alkali and alkaline earth metals (AAEMs) analysis of un-treated and glycerol pretreated corncobs.

Samples	AAEMs (mg/kg)					Normalized total AAEMs valencies
	Mg	Ca	Na	K	Total	
Un-treated	566.0	1185.2	82.4	4809.9	6643.5	—
220-0.5	294.8	993.3	40.5	190.1	1518.7	0.3
220-1	275.1	740.0	30.4	145.4	1190.9	0.3
220-2	274.7	482.0	17.7	120.2	894.6	0.2
220-3	274.9	325.6	17.7	82.9	701.1	0.2
240-0.5	306.5	802.3	39.8	182.8	1331.4	0.3
240-1	282.6	527.1	29.2	139.4	978.3	0.2
240-2	241.8	487.0	15.9	117.5	862.2	0.2
240-3	205.2	395.3	18.7	67.7	686.9	0.1
240-3-10	261.5	513.2	16.5	126.3	917.5	0.2
240-3-15	255.4	669.0	28.1	152.5	1105.0	0.2
240-3-20	282.0	823.2	24.2	138.8	1268.2	0.3

**Table 5:** Characteristic parameters of un-treated and glycerol pretreated corncobs. CrI: crystallinity index.

Samples	CrI (%)	T_i_ (°C)	T_max_ (°C)	DTG_max_ (%/°C)	Residue (%)
Un-treated	40.7	211.2	307.6	0.9	19.8
220-0.5	45.1	246.6	330.2	1.3	16.7
220-1	56.3	266.6	330.9	1.6	14.1
220-2	66.9	260.4	328.6	1.6	12.1
220-3	69.9	283.2	327.4	1.7	11.3
240-0.5	50.7	268.7	328.0	1.6	14.8
240-1	62.9	266.3	326.9	1.8	14.4
240-2	70.6	284.4	326.5	1.9	11.5
240-3	72.9	282.7	325.0	1.7	10.3
240-3-10	71.2	281.5	327.3	1.7	10.8
240-3-15	67.1	277.2	330.6	1.5	12.6
240-3-20	64.8	281.5	330.6	1.5	12.6

**Table 6:** Yields of main compounds from un-treated and glycerol pretreated corncobs in fast pyrolysis. 5-HMF: 5-hydroxymethylfurfural.

Samples	Acetic Acid	Furfural	5-HMF	Levoglucosan
Un-treated	7.8	0.8	0.4	2.2
220-0.5	6.6	0.6	0.4	10.4
220-1	5.8	0.6	0.4	20.0
220-2	4.6	0.5	0.5	27.5
220-3	3.9	0.4	0.6	30.3
240-0.5	6.1	0.6	0.5	19.3
240-1	4.3	0.5	0.5	26.8
240-2	3.1	0.5	0.6	33.0
240-3	2.9	0.4	0.7	35.8
240-3-10	3.2	0.5	0.7	33.1
240-3-15	3.8	0.6	0.6	30.7
240-3-20	4.4	0.6	0.6	28.6

## References

[1] Liu Y., Via B.K., Pan Y.F., Cheng Q.Z., Guo H.W., Auad M.L., Taylor S. (2017). Preparation and characterization of epoxy resin cross-linked with high wood pyrolysis bio-oil substitution by acetone pretreatment. Polymers.

[2] Binder J.B., Raines R.T. (2010). Fermentable sugars by chemical hydrolysis of biomass. Proc. Natl. Acad. Sci. USA.

[3] Rover M.R., Johnston P.A., Jin T., Smith R.G., Brown R.C., Jarboe L. (2014). Production of clean pyrolytic sugars for fermentation. ChemSusChem.

[4] Bailliez V., Olesker A., Cleophax J. (2004). Synthesis of polynitrogenated analogues of glucopyranoses from levoglucosan. Tetrahedron.

[5] Cao F., Schwartz T.J., McClelland D.J., Krishna S.H., Dumesic J.A., Huber G.W. (2015). Dehydration of cellulose to levoglucosenone using polar aprotic solvents. Energy Environ. Sci..

[6] Kitamura Y., ABE Y., Yasui T. (1991). Metabolism of levoglucosan (1,2-anhydro-β-d-glucopyranose) in Microorganisms. Agric. Biol. Chem..

[7] Nakagawa M., Sakai Y., Yasui T. (1984). Itaconic acid fermentation of levoglucosan. J. Ferment. Technol..

[8] Zhuang X.L., Zhang H.X. (2002). Identification, characterization of levoglucosan kinase, and cloning and expression of levoglucosan kinase cDNA from *Aspergillus. niger* CBX-209 in *Escherichia coli*. Protein Expr. Purif..

[9] Lian J.N., Garcia-Perez M., Chen S.L. (2013). Fermentation of levoglucosan with oleaginous yeasts for lipid production. Bioresour. Technol..

[10] Layton D.S., Ajjarapu A., Choi D.W., Jarboe L.R. (2011). Engineering ethanologenic *Escherichia coli* for levoglucosan utilization. Bioresour. Technol..

[11] Prosen E.M., Radlein D., Piskorz J., Scott D.S., Legge R.L. (1993). Microbial utilization of levoglucosan in wood pyrolysate as a carbon and energy source. Biotechnol. Bioeng..

[12] Bennett N.M., Helle S.S., Juff S.J.B. (2009). Extraction and hydrolysis of levoglucosan from pyrolysis oil. Bioresour. Technol..

[13] Luque L., Oudenhoven S., Westerhof R., Rossum G.V., Berruti F., Kersten S., Rehmann L. (2016). Comparison of ethanol production from corn cobs and switchgrass following a pyrolysis-based biorefinery approach. Biotechnol. Biofuels.

[14] Hosoya T., Kawamoto H., Saka S. (2007). Cellulose-hemicellulose and cellulose-lignin interactions in wood pyrolysis at gasification temperature. J. Anal. Appl. Pyrolysis.

[15] Zheng A.Q., Zhao Z.L., Huang Z., Zhao K., Wei G.Q., Jiang L.Q., Wang X.B., He F., Li H.B. (2015). Overcoming biomass recalcitrance for enhancing sugar production from fast pyrolysis of biomass by microwave pretreatment in glycerol. Green Chem..

[16] Jiang L.Q., Wu N.N., Zheng A.Q., Zhao Z.L., He F., Li H.B. (2016). The integration of dilute acid hydrolysis of xylan and fast pyrolysis of glucan to obtain fermentable sugars. Biotechnol. Biofuels.

[17] Jiang L.Q., Wu N.N., Zheng A.Q., Liu A.Q., Zhao Z.L., Zhang F., He F., Li H.B. (2017). Comprehensive utilization of hemicellulose and cellulose to release fermentable sugars from corncobs via acid hydrolysis and fast pyrolysis. ACS Susbtain. Chem. Eng..

[18] Sluiter A., Hames B., Ruiz R., Scarlata C., Sluiter B., Templeton D., Crocker D. (2008). Determination of Structural Carbohydrates and Lignin in Biomass.

[19] Segal L., Creely J.J., Martin A.E., Conrad C.M. (1959). An empirical method for estimation the degree of crystallinity of native cellulose using the X-ray diffractometer. Text. Res. J..

[20] Zambanini T., Kleineberg W., Sarikaya E., Buescher J.M., Meurer G., Wierckx N., Blank L.M. (2016). Enhanced malic acid production from glycerol with high-cell density *Ustilago. trichophora* TZ1 cultivations. Biotechnol. Biofuels.

[21] Zambanini T., Tehrani H.H., Geiser E., Merker D., Krabbe J., Buescher J.M., Meurer G., Wierckx N., Blank L.M. (2017). Efficient itaconic acid production from glycerol with *Ustilago. vetiveriae* TZ1. Biotechnol. Biofuels.

[22] Le Brech Y., Ghislain T., Leclerc S., Bouroukba M., Delmotte L., Brosse N., Snape C., Chaimbault P., Dufour A. (2016). Effect of potassium on the mechanisms of biomass pyrolysis studied using complementary analytical techniques. ChemSusChem.

[23] Wang Z.H., McDonald A.G., Westerhof R.J.M., Kersten S.R.A., Cuba-Torres C.M., Ha S., Pecha B., Garcia-Perez M. (2013). Effect of cellulose crystallinity on the formation of a liquid intermediate product distribution during pyrolysis. J. Anal. Appl. Pyrolysis.

[24] Jiang L.Q., Zheng A.Q., Zhao Z.L., He F., Li H.B., Wu N.N. (2016b). The comparison of obtaining fermentable sugars from cellulose by enzymatic hydrolysis and fast pyrolysis. Bioresour. Technol..

[25] Bridgwater A.V. (2012). Review of fast pyrolysis of biomass and product upgrading. Biomass Bioenergy.

[26] Hosoya T., Sakaki S. (2013). Levoglucosan formation from crystalline cellulose: Importance of a hydrogen bonding network in the reaction. ChemSusChem.

[27] Mukarakate C., Mittal A., Ciesielski P.N., Budhi S., Thompson L., Iisa K., Nimlos M.R., Donohoe B.S. (2016). Influence of crystal allomorph and crystallinity on products and behavior of cellulose during fast pyrolysis. ACS Susbtain. Chem. Eng..

[28] Piskorz J., Radlein D.S., Scott D.S., Czernik S. (1989). Pretreatment of wood and cellulose for production of sugars by fast pyrolysis. J. Anal. Appl. Pyrolysis.

[29] Fuentes M.E., Nowakowski D.J., Kubacki M.L., Cove J.M., Bridgeman T.G., Jones J.M. (2008). Survey of influence of biomass mineral matter in thermochemical conversion of short rotation willow coppice. J. Energy Inst..

[30] Wang K.G., Zhang J., Shanks B.H., Brown R.C. (2015). The deleterious effect of inorganic salts on hydrocarbon yields from catalytic pyrolysis of lignocellulosic biomass and its mitigation. Appl. Energy.

[31] Patwardhan P.R., Satrio J.A., Brown R.C., Shanks B.H. (2010). Influence of inorganic salts on the primary pyrolysis products of cellulose. Bioresour. Technol..

[32] Jarboe L.R., Liu P., Royce L.A. (2011). Engineering inhibitor tolerance for the production of biorenewable fuels and chemicals. Curr. Opin. Chem. Eng..

